# Rehabilitation for patients with retinal diseases in Brazil: An exploratory study

**DOI:** 10.5935/0004-2749.2024-0144

**Published:** 2024-10-31

**Authors:** Cecília Francini Cabral de Vasconcellos, Marina Leite Brandão, Juliana Maria Ferraz Sallum

**Affiliations:** 1 Instituto de Genética Ocular, São Paulo, SP, Brazil

**Keywords:** Rehabilitation, Quality of life, Retinal diseases, Personal satisfaction, Patient care team, Public policy, Surveys and questionnaires, Brazil

## Abstract

**Purpose:**

To evaluate the current scenario of rehabilitation services for people with retinal diseases in Brazil.

**Methods:**

An exploratory study was conducted between February 2023 and June 2023 using a Google Forms questionnaire that was distributed by patient associations.

**Results:**

A total of 142 patients, aged 18-80 were included in the study. Forty-eight participants (33.8%) were undergoing rehabilitation, while 94 (66.2%) were not. The main reason for not undergoing rehabilitation was a lack of knowledge about the service (n=41, 43.6%). Healthcare professionals made the most referrals (n=20, 41.7%). Rehabilitation improved the quality of life in 38 (80.9%) participants, and 28 (62.2%) participants were satisfied with the process. There was a statistically significant disparity between patient satisfaction and the locale of rehabilitation implementation. Twenty-three (69.7%) participants who underwent rehabilitation at a specialized center reported satisfaction.

**Conclusion:**

The rehabilitation process directly increases the quality of life of individuals with retinal diseases. However, despite the availability of rehabilitation centers in large parts of Brazil, most patients with retinal diseases are not acquainted with the rehabilitation process and do not receive referrals for it. Thus, healthcare providers should increase referrals to rehabilitation centers, and public policies should be formulated to raise awareness among the population regarding the availability of rehabilitation services.

## INTRODUCTION

Retinal diseases are one of the leading causes of visual impairment from childhood to old age. These can include common conditions such as age-related macular degeneration, diabetic retinopathy, and retinopathy of prematurity or rare conditions such as inherited retinal dystrophies^(1)^.

In addition to developing visual impairment, people with retinal diseases also experience depression, anxiety, and a poor quality of life^(2^,^3)^. These patients and their families might also experience educational and financial difficulties. Thus, a retinal disease can directly impact the patient’s well-being costs, health services, and productivity^(4^-^7)^.

In ophthalmology, visual impairment has been traditionally determined by assessing visual acuity and visual field. However, the current understanding of disability is that it results from the interaction between the individual’s condition and societal barriers. This new definition is based on the Convention on the Rights of Persons with Disabilities of the United Nations (UN), and it was incorporated into Brazilian law on August 25, 2009, by Decree No. 6949 ^(8^,^9)^.

Considering the new concept of disability and the perception of the biopsychosocial impact of retinal diseases, rehabilitation appears to be crucial for improving the care and quality of life of such patients. Rehabilitation is an interdisciplinary and multiprofessional approach that aims to teach individuals to adapt or readapt to their disability. In doing so, the patients can achieve their full cognitive, behavioral, and functional potential. By learning to deal with and adapt to their condition, individuals with visual impairment can return to their daily activities. Moreover, these individual become more independent and autonomous^(2^,^10)^.

In 2017, the World Health Organization (WHO) declared rehabilitation as a health strategy for the 21st century^(9)^. However, although the benefits of rehabilitation have been established, referrals to these centers remain insufficient. In the study by Coker et al.^(11)^, only 11.4% of the 143 patients with visual impairment who were evaluated at an ophthalmology clinic in Alabama were referred for rehabilitation.

According to the World Report on Disability^(12)^, rehabilitation centers lack physical, attitudinal, and institutional accessibility. These centers present substantial barriers such as negative attitudes of the professionals toward users, inadequate technologies and formats of information and communication, and lack of participation of patients in the decision-making about their rehabilitation^(13)^.

A systematic review that evaluated rehabilitation in developing countries concluded that only a few studies exist in this field and that people with disabilities have limited access to rehabilitation^(14)^. Another study in Brazil revealed that the access to rehabilitation for individuals with disabilities is low. Furthermore, this was mainly observed at primary health care centers, where predominantly socioeconomically disadvantaged individuals are served^(15)^.

If rehabilitation plays an essential role in social inclusion and is a right registered by the UN convention, more studies should be conducted on this topic. Thus, in this study, we aimed to investigate the current scenario of rehabilitation services for people with retinal diseases in Brazil.

## METHODS

This exploratory cross-sectional study was conducted in accordance with the tenets of the World Medical Association’s Declaration of Helsinki on Ethical Principles for Medical Research Involving Human Subjects. This study was approved by the Research Ethics Committee of *Hospital Olhos Paulista* (No: 5.797.733; |december 06, 2022|).

### Sample

The study sample was obtained via convenience sampling on the basis of the criterion of theoretical saturation. The study included 142 individuals with visual impairment due to retinal diseases, who were aged 18-80 years and willing to fill out a Google Forms questionnaire. People with visual impairment due to other causes were excluded from the study.

### Instruments

A questionnaire with close-ended questions was prepared using Google Forms and used as the assessment tool in this study. The questions aimed to determine the current rehabilitation scenario for people with retinal diseases in Brazil. The questionnaire included the following aspects: participant’s age, rare or common retinal disease, time of diagnosis, rehabilitation status, reason for not undergoing rehabilitation, interval between diagnosis and rehabilitation, format of rehabilitation, rehabilitation actions, distance between the rehabilitation center and the participant’s residence, proponent of the referral to rehabilitation, improvement in the quality of life after rehabilitation, and satisfaction with rehabilitation.

### Procedures

The questionnaire was forwarded to the patient groups of Retina Brazil via WhatsApp from February 2023 to June 2023. Patients interested in participating in the study provided informed consented before completing the questionnaire, which took approximately 7 minutes.

### Data processing and analysis

The data were extracted onto a Google Forms spreadsheet and compiled and analyzed using R statistical software^(16)^. Descriptive statistical analyses were performed, and the data are presented as means and standard deviation. The Shapiro-Wilk test was used to verify normal distribution of the data. Fisher’s exact test was used to study the relationships between the categorical variables. A p-value of <0.05 was considered statistically significant.

## RESULTS

A total of 142 individuals with retinal diseases, aged 18-80 years, participated in this study. Of the 142 individuals, 30 (20.4%) were aged 18-35 years, 57 (40.1%) were aged 35-55 years, and 56 (39.4%) were aged 55-80 years. Of these participants, 107 (75.9%) had rare diseases and 34 (24.1%) had common conditions. In this study, 48 (33.8%) patients were undergoing rehabilitation and 94 (66.2%) were not. The patients’ diagnostic time ranged from 0 to ≥10 years. The diagnostic time was 0-1 year in 10 (7%) patients, 1-5 years in 34 (23.9%) patients, 5-10 years in 25 (17.6%) patients, and ≥10 years in 73 (51.4%) patients. The interval between diagnosis and rehabilitation also varied from 0 to ≥10 years. The interval was 0-1 year in 16 (33.3%) patients, 1-5 years in 8 (16.7%) patients, 5-10 years in 3 (6.2%) patients, and ≥10 years in 7 (14.6%) patients. Tables 1, 2, and 3 present the rehabilitation format undertaken by the participants, distances between the rehabilitation centers and participants’ residences, and main individuals responsible for the referral for rehabilitation, respectively.

**Table 1:** Rehabilitation formats determined in the study

Rehabilitation Center n (%)	Mixed n (%)	Private Tutors n (%)	Alone n (%)
35 (72.9)	3 (6.2)	3 (6.2)	7 (14.6)

**Table 2:** Distance between the rehabilitation center and the participant’s residence

Located in my city n (%)	Located in my neighborhood n (%)	Located in my state n (%)	Not near my residence n (%)
15 (38.5)	3 (7.7)	9 (23.1)	12 (30.8)

**Table 3:** Individuals responsible for the referral to rehabilitation centers

Friends n (%)	Association/Group n (%)	Relatives n (%)	Mixed n (%)	Healthcare providers n (%)	Rehabilitation sought out by the participant n (%)
6 (12.5)	4 (8.3)	2 (4.2)	11 (22.9)	20 (41.7)	5 (10.4)

Table 4 presents the main reasons for not undergoing rehabilitation. Table 5 presents the most common activities undertaken in the rehabilitation program.

**Table 4:** Reason for not undergoing rehabilitation

No need n (%)	Wasn’t aware of its existence n (%)	Wasn’t aware of its existence and not near my home n (%)	Not near my home n (%)	Never wanted to n (%)	Other n (%)
12 (12.8)	41 (43.6)	9 (9.6)	23 (24.5)	5 (5.3)	4 (4.3)

**Table 5:** Percentage of rehabilitation actions undertaken by the individuals

Which rehabilitation actions did you undertake?	Frequency n (%)
Orientation and mobility	35 (72.9)
Psychological support	26 (54.2)
Training for the use of electronics	24 (50)
Optical aids	21 (43.8)
Daily life activities (e.g., cooking and tidying the house)	20 (41.7)
Braille	15 (31.2)
Other	13 (27.1)

The questionnaire results revealed that 38 (80.9%) patients agreed that rehabilitation improved their quality of life. However, 1 (2.1%) patient disagreed and 8 (17%) patients neither agreed nor disagreed that rehabilitation improved their quality of life. There was no statistically significant correlation between the rehabilitation format and the improvement in the quality of life (p=0.159). The questionnaire results revealed that 28 (62.2%) patients were satisfied with the rehabilitation, while 6 (13.3%) were not. However, 11 (24.4%) patients were neither satisfied nor dissatisfied with the rehabilitation. Furthermore, there was a statistically significant correlation between the rehabilitation format and patient satisfaction (p=0.011; Table 6). Moreover, most participants (n=23, 69.7%) were satisfied with the rehabilitation services provided at a rehabilitation center.

**Table 6:** Correlation between rehabilitation format and satisfaction

	Disagree n (%)	Neither agree nor disagree n (%)	Agree n (%)	Total n (%)
Rehabilitation center	1 (3)	9 (27.3)	23 (69.7)	33 (73.3)
Mixed	1 (33.3)	1 (33.3)	1 (33.3)	3 (6.7)
Private tutors	1 (33.3)	1 (33.3)	1 (33.3)	3 (6.7)
Alone (he/she/they)	3 (50)	0 (0)	3 (50)	6 (13.3)
Total	6 (13.3)	11 (24.4)	28 (62.2)	45 (100)

There was no statistically significant correlation between the diagnosis-to-rehabilitation interval and the improvement in quality of life (p=0.967). Similarly, there was no statistically significant correlation between the diagnosis-to-rehabilitation interval and patient satisfaction (p=0.622).

There was no statistically significant correlation between individuals who received psychological support and an improved quality of life (p=0.561). However, a greater agreement that rehabilitation improves the quality of life was observed in patients who received psychological support than in those who did not (Table 7). Although there was also no statistically significant corre-lation between patient satisfaction with rehabilitation and psychological support (p=0.184), patients who received psychological support were more satisfied with the rehabilitation than those who did not receive psychological support (Table 8).

**Table 7:** Correlation between psychological support and quality of life

	Disagree n (%)	Neither agree nor disagree n (%)	Agree n (%)	Total n (%)
No	1 (4.8)	4 (19)	16 (76.2)	21 (44.7)
Yes	0 (0)	4 (15.4)	22 (84.6)	26 (55.3)
Total	1 (2.1)	8 (17)	38 (80.9)	47 (100)

**Table 8:** Correlation between psychological support and patient satisfaction

	Disagree n (%)	Neither agree nor disagree n (%)	Agree n (%)	Total n (%)
No	1 (5)	7 (35)	12 (60)	20 (44.4)
Yes	5 (20)	4 (16)	16 (64)	25 (55.6)
Total	6 (13.3)	11 (24.4)	28 (62.2)	45 (100)

## DISCUSSION

The age of the participants in this study ranged from 18 to 80 years, without a predominant age group. This may be attributed to the fact that most of the study participants had rare retinal diseases. Rare retinal diseases can manifest from the first year of life and have a broad spectrum of symptoms and severity^(17^-^19)^.

Most individuals with visual impairments do not participate in rehabilitation^(15)^. This study’s sample also reveals a low participation (33.8%). However, a significant portion of the individuals had a rehabilitation center in their neighborhood, city, or state (69.3%). This is in contrast with the finding of previous studies that reported a scarcity of rehabilitation centers across different regions of Brazil^(15^,^20^-^23)^.

In our study, the main reason for patients not undergoing rehabilitation was a lack of knowledge about the process (43.6%). This indicates that even if there are centers near a patient’s residence, they might be unaware of them. Therefore, these centers are not being utilized.

The diagnostic time in our study varied significantly, ranging from 0 to ≥10 years. The interval between diagnosis and rehabilitation was also diverse. Sixteen (33.3%) patients underwent rehabilitation within one year of being diagnosed, while 16 (33.3%) patients underwent rehabilitation after >10 years. This indicates that there may be no relationship between the diagnostic time and rehabilitation. Additionally, the interval between diagnosis and rehabilitation does not influence the improvement in quality of life or patient satisfaction.

Most of the study participants (72.9%) underwent rehabilitation at specialized centers and were satisfied with the process. Furthermore, there was a statistically significant correlation between patient satisfaction and do rehabilitation in a rehabilitation center (p=0.011). However, there was no statistically significant corre-lation between the improvement in quality of life improvement and rehabilitation format. This indicates that even if patient satisfaction varies with the type of rehabilitation, any form of rehabilitation improves the quality of life.

The patient satisfaction in our study differs from that of previous studies, which reported dissatisfaction with rehabilitation centers due to issues such as lack of accessibility and equity and discrimination against patients^(21^-^23)^. This study did not compare the satisfaction with public and private rehabilitation centers, which could have affected the results. Private rehabilitation centers tend to offer more comprehensive hours with complete multiprofessional and interdisciplinary teams^(20^,^24)^.

The primary agents referring participants to rehabilitation centers in our study were health teams (41.7%). This may be associated with the growing emphasis on protocols for communicating bad news in ophthalmology. In ophthalmology, a disease without a cure or treatment that leads to visual impairment is considered bad news^(25^,^26)^. One of the protocols used in bad news communication is SPIKES^(25^,^26)^, which divides the communication of bad news into stages. The last stage of this protocol is related to strategies and summaries, in which coping strategies to deal with the bad news are established. Referring agents typically suggest rehabilitation during these moments. This stage is crucial in helping patients feel supported and confident to face their condition^(27^,^28)^.

According to most of our study participants (80.9%), rehabilitation improved their quality of life. Individuals with visual impairments and retinal diseases tend to have a lower quality of life and significant emotional health concerns. Thus, any improvement in the quality of life in this population is significant. This study’s findings differ from those of previous studies that have suggested that rehabilitation does not improve quality of life^(29)^. Similarly, in a systematic review by Van Nispen et al.^(30)^, there are no robust data indicating improvements in the health or quality of life in individuals with visual impairment who participated in rehabilitation and psychotherapy groups.

Previous studies have concluded that there are no robust findings to prove that psychological treatments improvement a patient’s quality of life^(30)^. Similarly, we did not find a statistically significant correlation between psychological support and patient satisfaction or improvement in quality of life in our study. However, both patient satisfaction and improvement in quality of life were more prevalent in participant who were provided psychological support.

A limitation of this study was the distinct number of patients with rare and common diseases. Thus, it was challenging to discern the differences between the groups. Moreover, a few people who underwent rehabilitation could not comment on their experience. Future studies with larger patient samples who have undergone rehabilitation are required to better understand this process and make more accurate correlations. Further studies with specific psychotherapy protocols for individuals with visual impairment need to be conducted to better understand the effect of psychological support in this population.

Rehabilitation plays a fundamental role in improving an individual’s quality of life, regardless of the interval between diagnosis and its implementation. Thus, it is crucial to increase the referrals of this population to rehabilitation centers. Although rehabilitation centers are available in most cities or states, people with retinal diseases are often unaware of the process and its psychosocial impacts. Thus, promoting the benefits of rehabilitation may be a key factor in improving its utilization.

Rehabilitation centers must be prepared for various rehabilitation modalities according to their users need. A more comprehensive rehabilitation process will ensure greater improvements in the user’s autonomy and independence. Most users commended the quality of services provided at rehabilitation centers, indicating that the existing centers provide a high standard of care. Thus, these centers could be benefit from government policies that support the expansion of the services offered and facilitate the establishment of additional rehabilitation facilities.
